# Fibulin7 Mediated Pathological Cardiac Remodeling through EGFR Binding and EGFR‐Dependent FAK/AKT Signaling Activation

**DOI:** 10.1002/advs.202207631

**Published:** 2023-06-21

**Authors:** Xuehui Zheng, Lingxin Liu, Jing Liu, Chen Zhang, Jie Zhang, Yan Qi, Lin Xie, Chunmei Zhang, Guoqing Yao, Peili Bu

**Affiliations:** ^1^ The Key Laboratory of Cardiovascular Remodeling and Function Research Chinese Ministry of Education Chinese National Health Commission and Chinese Academy of Medical Sciences The State and Shandong Province Joint Key Laboratory of Translational Cardiovascular Medicine Department of Cardiology Qilu Hospital Cheeloo College of Medicine Shandong University Jinan 250012 China; ^2^ Department of Cardiology Heze Municipal Hospital Heze 274000 China

**Keywords:** EGFR, fibrosis, fibulin 7, myocardial infarction, myofibroblasts

## Abstract

Adverse remodeling after myocardial infarction (MI) result in heart failure and sudden cardiac death. Fibulin7 (FBLN7) is an adhesion protein excreted into the extracellular matrix that functions in multiple biological processes. However, whether and how FBLN7 affects post‐MI cardiac remodeling remains unclear. Here, the authors identify FBLN7 as a critical profibrotic regulator of adverse cardiac remodeling. They observe significantly upregulated serum FBLN7 levels in MI patients with left ventricular remodeling compared to those without MI. Microarray dataset analysis reveal FBLN7 is upregulated in human heart samples from patients with dilated and hypertrophic cardiomyopathy compared with non‐failing hearts. The authors demonstrate that FBLN7 deletion attenuated post‐MI cardiac remodeling, leading to better cardiac function and reduced myocardial fibrosis, whereas overexpression of FBLN7 results in the opposite effects. Mechanistically, FBLN7 binds to the epidermal growth factor receptor (EGFR) through its EGF‐like domain, together with the EGF‐like calcium‐binding domain, and induces EGFR autophosphorylation at tyrosine (Y) 1068 and Y1173, which activates downstream focal adhesion kinase/AKT signaling, thereby leading to fibroblast‐to‐myofibroblast transdifferentiation. In addition, FBLN7‐EGFR mediates this signal transduction, and the fibrotic response is effectively suppressed by the inhibition of EGFR activity. Taken together, FBLN7 plays an important role in cardiac remodeling and fibrosis after MI.

## Graphical abstract

FBLN7 is secreted after MI and binds to EGFR via its EGF‐like and EGF‐CA domains that activate EGFR kinase activity and the subsequent intracellular FAK/AKT signaling pathway, which conveys abnormal EGFR signals from the CF surface to the nucleus, thereby leading to myofibroblast hyperactivation and ultimately cardiac fibrosis. These signal transductions and fibrotic responses could be effectively inhibited by Gefitinib.

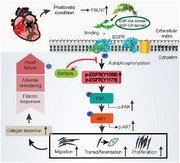

## 1 Introduction

Adverse structural remodeling of the left ventricle due to myocardial infarction (MI) is a common pathological feature of heart failure (HF).^[^ ^1^ ^]^ It is characterized by focal fibrotic scar formation and compensatory diffuse myocardial fibrosis in remote cardiac tissue. Cardiac fibrosis is characterized by the excessive deposition of collagen‐rich extracellular matrix (ECM),^[^ ^2^ ^]^ a fundamental process observed in multiple cardiovascular diseases.^[^ ^3^ ^]^ It is now accepted that alterations in the ECM result in cardiac fibrosis and lead to HF.^[^ ^4^, ^5^ ^]^ Cardiac fibroblasts (CFs), which phenotypically transform into myofibroblasts in response to heart injury, play a central role in the pathophysiology consequences of MI.^[^ ^6^ ^]^ Specifically, necrotic myocytes are replaced by focal collagen‐rich scars during the early stage, and progressive fibrosis in the non‐infarct area occurs during the chronic stage because of sustained fibroblast activation.^[^ ^7^ ^]^ Suppressing the hyperactivation of CFs in the late phase of MI can ameliorate myocardial fibrosis;^[^ ^7^, ^8^ ^]^ however, the underlying mechanisms are not fully understood.

The fibulin family is a group of eight ECM glycoproteins involved in tissue remodeling, cell‐matrix interactions, and the conversion of cell phenotypes.^[^ ^9^, ^10^ ^]^ Fibulin 7 (FBLN7) is a newly identified member of the fibulin family and is expressed in the eye, placenta, cartilage, teeth, blood vessels, and other vital tissues.^[^ ^11^ ^]^ It is involved in multiple diseases and developmental anomalies,^[^ ^12^ ^]^ such as 2q13 deletion syndrome, glioblastoma, renal tubular calcification, and breast tumors. Moreover, as an adhesion protein, FBLN7 has been shown to affect biological processes such as angiogenesis,^[^ ^13^ ^]^ cell spreading and migration,^[^ ^14^ ^]^ and immunoregulation^[^ ^15^, ^16^ ^]^ via interactions with integrins,^[^ ^15^ ^]^ heparin sulfate receptors,^[^ ^17^ ^]^ or vascular endothelial growth factor receptor 2^[^ ^18^ ^]^ located on the cell surface. It is conceivable that FBLN7 can also modulate the properties of fibroblasts, as it colocalizes with fibronectin during early development. However, there has been little discussion of this aspect.

Several fibulins are involved in the regulation of tissue remodeling and fibrosis. For instance, fibulin 1(FBLN1) participates in vascular remodeling^[^ ^19^ ^]^ and pulmonary fibrosis;^[^ ^20^, ^21^ ^]^ fibulin 2 (FBLN2) is involved in cardiac fibrosis;^[^ ^22^ ^]^ fibulin 3 (FBLN3) participates in vascular remodeling;^[^ ^23^ ^]^ and fibulin 5 is associated with cutaneous fibrosis^[^ ^24^ ^]^ and hepatic fibrosis.^[^ ^25^ ^]^ Due to their similar amino acid sequences and domain structures, members of the fibulin family are likely to exhibit comparable biological functions. Thus, FBLN7 is likely involved in cardiac remodeling and fibrosis. However, there is no experimental evidence to support this hypothesis.

Defining the mechanisms involved in CF hyperactivation is critical for understanding the regulation of post‐MI myocardial fibrosis, which is essential for preventing adverse cardiac remodeling and HF. Therefore, our study aimed to explore the role of FBLN7 in fibroblast activation and cardiac fibrosis using a mouse model of MI, with a special emphasis on revealing the explicit molecular mechanisms and specific functional motifs of FBLN7. We hope that this study will contribute to a deeper understanding of the pathogenesis of myocardial fibrosis and fill the gap in our understanding of FBLN7.

## 2 Results

### 2.1 FBLN7 is Involved in Cardiac Fibrosis

**Figure 1:**
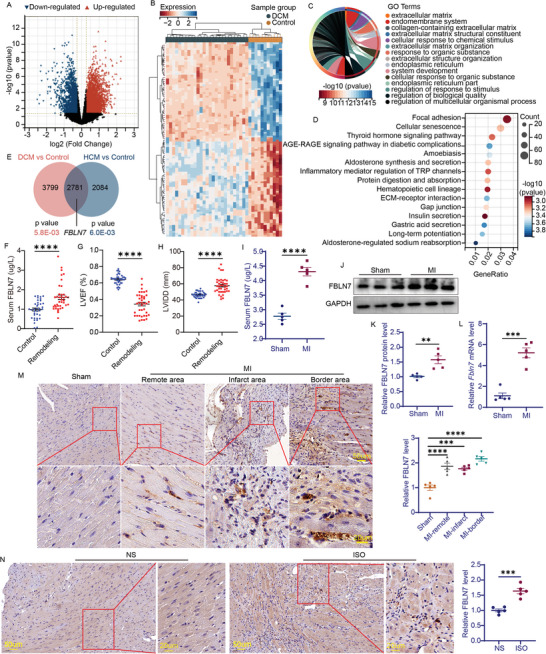
FBLN7 is upregulated in pathologically remodeled heart. A–E) Bioinformatics analysis based on differentially expressed genes (DEGs) between the myocardial samples from dilated cardiomyopathy (*n* = 27) and those from “normal” donors (*n* = 11) in the publicly available microarray dataset (GSE1145) from the Gene Expression Omnibus database. A) Volcano plot of all DEGs (|fold change|> 1.2, *p*<0.05). B) Hierarchical clustering heatmap and dendrogram of samples based on top 50 DEGs. Genes are clustered by row, and samples by column. C) Top 15 enriched gene ontology terms (FDR<0.1, *p*< 0.05). D) Top 15 enriched Kyoto Encyclopedia of Genes and Genomes pathways (FDR<0.1, *p*< 0.05). E) Overlap of DEGs between the dilated cardiomyopathy versus “normal” samples and hypertrophic cardiomyopathy (*n* = 5) versus “normal” samples. F) ELISA detection of serum FBLN7 levels and echocardiography measurements of left ventricular ejection fraction G) and left ventricular internal diameter at diastole H) in myocardial infarction (MI) patients with cardiac remodeling (*n* = 39) and in those without MI (*n* = 37). I–M) Detections of FBLN7expression in wild‐type mice 28 days after MI or sham operation. Five mice were in each group. I) ELISA detection of serum FBLN7 levels. J and K) Representative western blot images and quantification of FBLN7 protein levels in the heart. L) Quantitative real‐time PCR analysis of Fbln7 mRNA expression in the heart. M) Representative images of immunohistochemical FBLN7 staining in sham‐operated hearts and in the infarct, border, and remote zones in infarcted hearts, quantified at the right. N) Representative images of immunohistochemical staining of FBLN7 in heart tissues of mice treated with sterile saline and isoproterenol. Quantification is shown on the right. Error bars are SEM. (F) by Mann–Whitney U test, (G–L,N) by Student t test, (M) by one‐way ANOVA followed by Dunnett post–hoc test. *p<0.05, ^**^ *p*<0.01, ^***^ *p*<0.001, and ^****^ *p*<0.0001.

To search for targets and molecular mechanisms underlying cardiac fibrosis related to HF, a publicly available microarray dataset, GSE1145, obtained from the Gene Expression Omnibus database, was analyzed. Compared with the myocardial samples from “normal” organ donors (control group, *n* = 11), samples from the dilated cardiomyopathy (DCM) group (*n* = 27) had 6580 differentially expressed genes (DEGs) (4689 upregulated and 1891 downregulated) (**Figure** **1**A), and those from the hypertrophic cardiomyopathy (HCM) group (*n* = 5) had 4865 DEGs (2903 upregulated and 1962 downregulated) (|fold change| > 1.2, *p* < 0.05) (Figure S1A, Supporting Information). Among them, the gene encoding the matricellular protein FBLN7, which was upregulated in both DCM and HCM groups, caught our attention (Figure 1E). To explore the possibility of FBLN7 involved in disease progression, DEGs were subjected to Gene Ontology (GO) enrichment analysis and Kyoto Encyclopedia of Genes and Genomes (KEGG) pathway enrichment analysis. GO analysis revealed that the DEGs between the DCM and control groups were significantly enriched in the ECM and involved in biological processes, including cellular response to chemical stimuli, ECM organization, and regulation of multicellular organismal processes (Figure 1C). KEGG analysis revealed significant enrichment of the focal adhesion pathway and its related ECM‐receptor interaction pathway (Figure 1D). Subsequently, GO enrichment and KEGG pathway analyses of DEGs between the HCM and control groups also indicated that terms and pathways associated with cell‐ECM interactions, such as cellular response to chemical stimulus, DCM pathway, and HCM pathway, were significantly enriched (Figure S2B,D, Supporting Information). From the above functional analyses, the regulation of DEGs involved in cell‐ECM interactions is evident as a potential mechanism for cardiac fibrosis. Based on information available in the UniProt and GeneCards databases, FBLN7 is localized in the ECM and is involved in cell adhesion. Therefore, we speculate that FBLN7 may be related to cardiac fibrosis.

Given that FBLN7 is a secreted protein, serum FBLN7 levels were measured in patients with post‐MI cardiac remodeling and in those without MI (control group). The baseline patient information is shown in Table S1 (Supporting Information). Serum FBLN7 levels were significantly higher in patients with post‐MI cardiac remodeling than in the control group, accompanied by a lower left ventricular fraction (LVEF) and a larger left ventricular internal diameter at diastole (LVIDD) (Figure 1F–H). Serum FBLN7 levels were significantly associated with lower LVEF (Pearson = −0.631) and larger LVIDD (Pearson = 0.606) in patients with post‐MI cardiac remodeling. Clinical data further suggested that FBLN7 may be associated with cardiac fibrosis.

### 2.2 FBLN7 is Upregulated in Mouse Models of Cardiac Fibrosis

We explored whether FBLN7 expression was altered in mouse models of cardiac fibrosis. Compared to the sham group, FBLN7 was gradually upregulated after MI and remained at a high level on day 28 (Figure S2A, Supporting Information). Based on this finding and previous reports,^[^ ^26^ ^]^ mice at day 28 post‐MI were used to explore the effects of FBLN7 on cardiac fibrosis and the underlying mechanisms. Consistent with clinical findings, serum FBLN7 levels detected by enzyme‐linked immunosorbent assay (ELISA) were also higher in post‐MI mice than in sham‐operated controls (Figure 1I). Compared with the weak FBLN7 signal observed in the sham‐operated group, immunohistochemistry (IHC) analysis showed that FBLN7 protein expression increased after MI but was unevenly distributed across the heart, with the strongest expression in the peri‐infarct area, weaker expression in remote areas, and the weakest expression in the infarct area (Figure 1 M). Upregulated FBLN7 was largely localized in the interstitial space but rarely in cardiomyocytes (Figure S2B, Supporting Information). The non‐infarcted area (including border and remote areas) of the myocardium from the MI mice and the myocardium from the sham mice were collected to measure FBLN7 protein (Figure 1J,K) and Fbln7 mRNA levels (Figure 1L), and consistent results were observed.

Similar findings were observed in a mouse model of isoproterenol‐induced cardiac fibrosis. IHC staining showed an increase in FBLN7 expression in the hypertrophic myocardium (Figure 1N), accompanied by an enlarged left ventricle and aggravated cardiac fibrosis, compared with the normal saline group (Figure S2C,D, Supporting Information). These findings support the hypothesis that FBLN7 expression is associated with cardiac fibrosis.

### 2.3 FBLN7 Deletion Ameliorates Post‐MI Cardiac Fibrosis and Improves Cardiac Function

**Figure 2:**
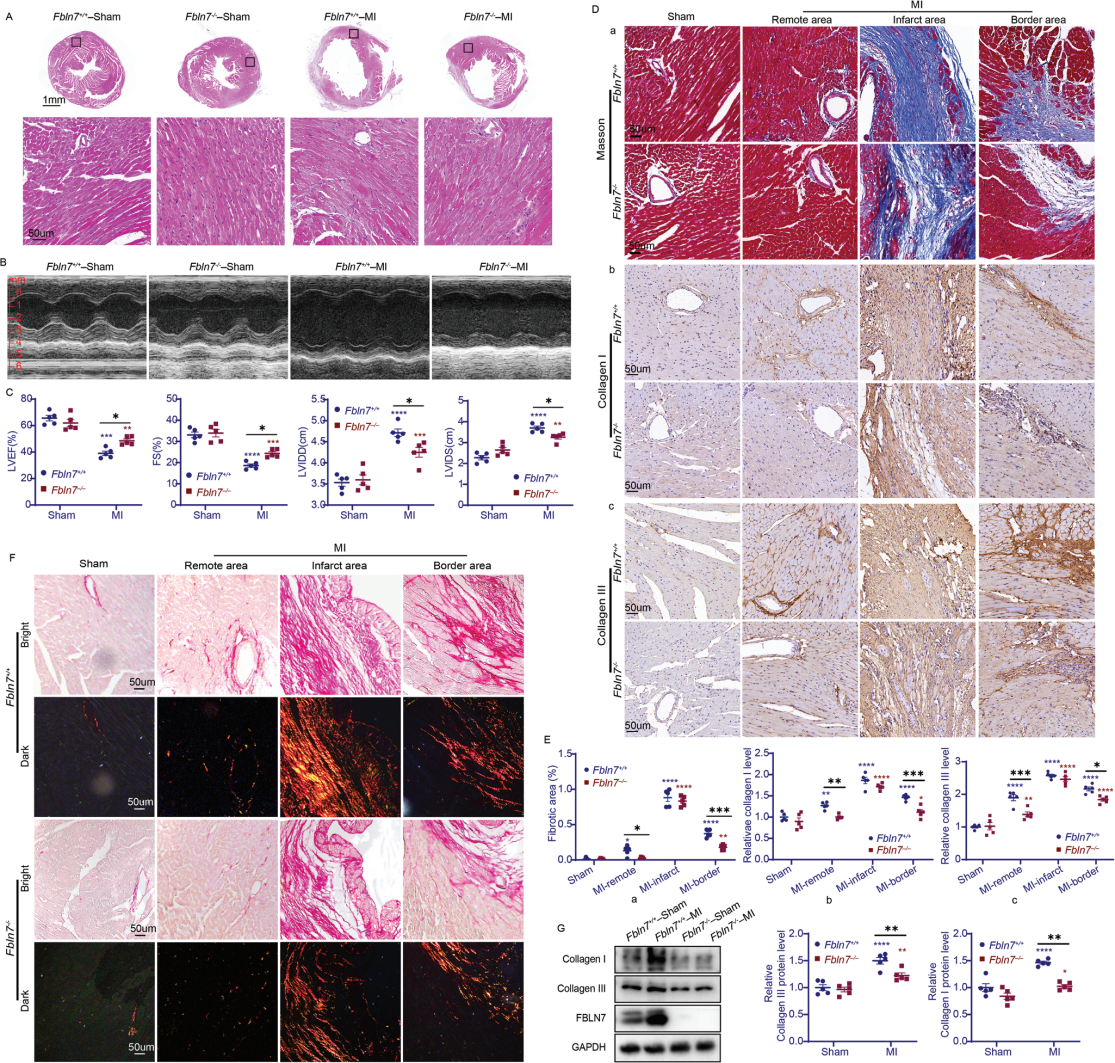
FBLN7 deletion protects mice against MI‐induced cardiac fibrosis. Cardiac function and remodeling were assessed in wild‐type (Fbln7^+/+^) and FBLN7‐KO (Fbln7^−/−^) mice 28 days after myocardial infarction (MI) or sham operation. A) Representative images of H&E staining of the hearts from the four groups of mice. Bottom: magnification of the boxed region. B) Representative M‐mode echocardiography images of the four groups of mice. C) Echocardiographic analysis of cardiac dimensions and function. D,E) Representative micrographs of Masson staining (D, panel a), and IHC staining for Collagen I (D, panel b) and III (D, panel c) in heart sections from the Fbln7^+/+^ and Fbln7^−/−^ mice following MI or sham operation. Three different zones (infarct, border, and remote) in infarcted hearts were displayed. Quantifications of fibrotic area and levels of collagen I and III were shown in (E). F) Representative Sirius red‐stained heart sections. G) Representative western blot images and quantifications of FBLN7, collagen I, and collagen III protein levels. Error bars are SEM. *p<0.05, ^**^ *p*<0.01, ^***^ *p*<0.001, and ^****^ *p*<0.0001 by two‐way ANOVA followed by Sidak post‐hoc test. Red asterisk, versus Fbln7^+/+^‐sham group. Blue asterisk, versus Fbln7^−/−^‐sham group.

FBLN7‐knockout (Fbln7^−/−^) mice do not show obvious developmental or health defects seen in the absence of other fibulin genes in animal models, such as bleeding,^[^ ^27^ ^]^ retinopathy,^[^ ^28^ ^]^ macular dystrophy,^[^ ^29^ ^]^ and defective elastic fiber formation.^[^ ^30^ ^]^ Wild‐type (Fbln7^+/+^) and Fbln7^−/−^ mice were randomly subjected to MI surgery or sham operation. Successful modeling was confirmed by hematoxylin and eosin (H&E) staining (**Figure** **2**A) and echocardiography (Figure 2B) 28 days after the operation. A thin fibrotic scar replaced the normal myocardium of the left ventricular (LV) wall in MI mice, and clear cardiac remodeling was demonstrated, as evidenced by an increased LVIDD and left ventricular internal diameter at systole (LVIDS) and a reduced LVEF and percent of fractional shortening (FS%) (Figure 2C). Excessive deposition of collagen I and III and myocardial fibrosis were also observed in the infarct and border areas of Fbln7^+/+^ hearts after MI (Figure 2D–G).

In contrast to the Fbln7^+/+^ group, the Fbln7^−/−^ group displayed a higher LVEF and FS% and a smaller LVIDD and LVIDS after MI (Figure 2B,C). Masson's trichrome (Figure 2D,E, panel a) and Sirius red staining (Figure 2F) showed less post‐MI fibrosis in Fbln7^−/−^ mice compared with Fbln7+/+ mice, and the difference was pronounced in the border and remote areas. Consistently, MI mice in the Fbln7^−/−^ group (Fbln7^−/−^‐MI) displayed less collagen I and III deposition than those in the Fbln7^+/+^ group (Fbln7^+/+^‐MI), with this difference being more pronounced in the non‐infarct area (Figure 2D,E, panel b,c). This was consistent with the changes in collagen I and III protein levels detected by western blot (WB) (Figure 2G). These data indicated that FBLN7 deletion mitigated adverse remodeling after MI in mice, attenuating myocardial fibrosis, LV dilatation, and cardiac dysfunction. In addition, there was no significant increase in mortality or LV rupture in the Fbln7^−/−^‐MI compared with the Fbln7^+/+^‐MI group, suggesting that FBLN7 deletion could ameliorate post‐MI adverse remodeling without impairing infarct healing.

### 2.4 Overexpression of FBLN7 Aggravates MI‐Induced Cardiac Dysfunction and Fibrosis

**Figure 3:**
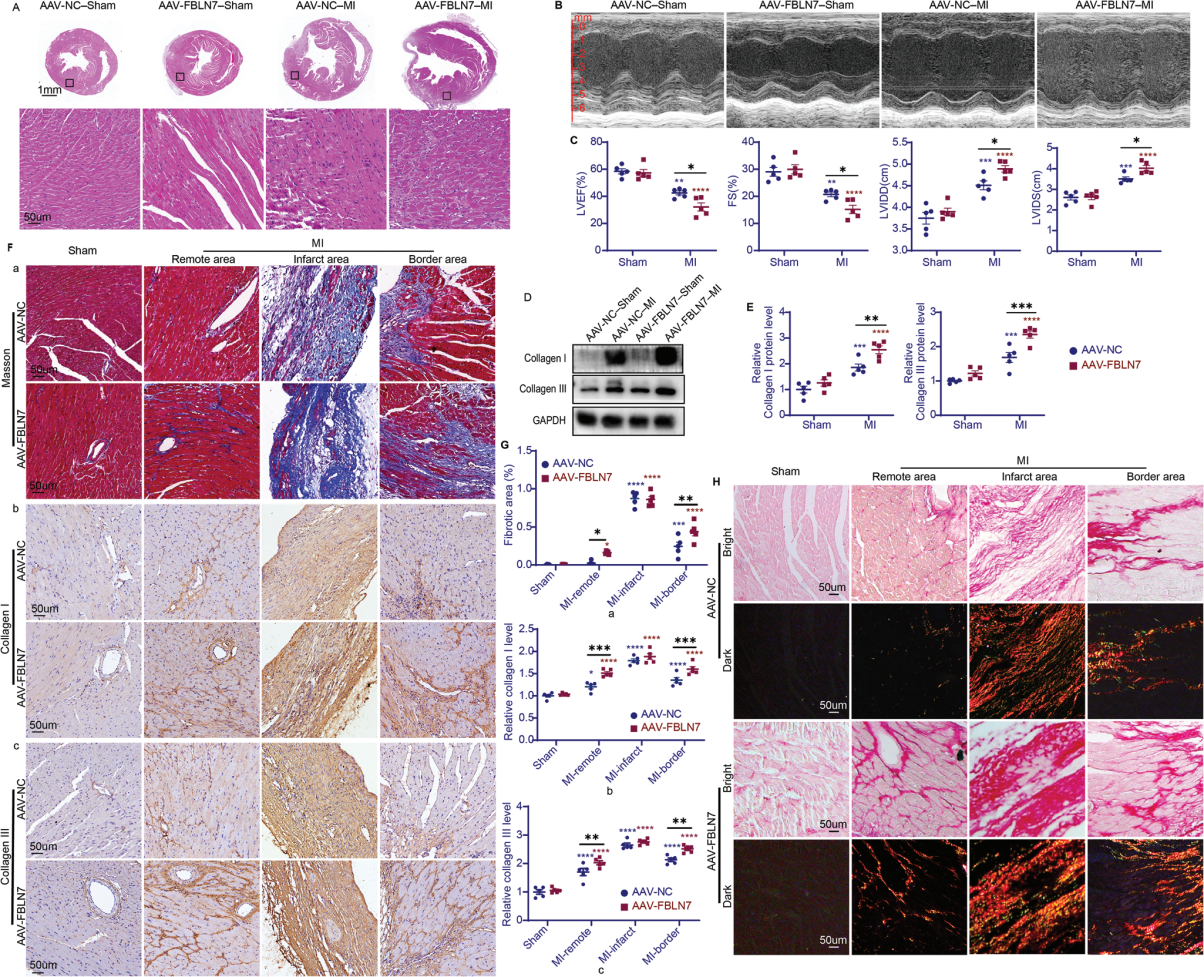
FBLN7 overexpression in the heart leads to a more profound fibrotic response following MI. Mice injected with adeno‐associated virus serotype 9 encoding FBLN7 (AAV‐FBLN7) or negative control (AAV‐NC) were randomly allocated into myocardial infarction (MI) group and sham operation group. Five mice in each group. A) Representative images of H&E staining of hearts from the four mouse groups. Bottom panel: magnification of the boxed region. B) Representative M‐mode echocardiograms from the four experimental groups. C) Echocardiographic analysis of left ventricular ejection fraction, percent of fractional shortening, left ventricular internal diameter at systole and at diastole. D,E) Representative western blot images of FBLN7, collagen I and III proteins from the four mouse groups, and quantitative analysis (E). F,H) Representative Masson staining (F, panel a), IHC staining for collagen I (F, panel b) and III (F, panel c), and Sirius Red staining (H) of heart sections from the four mice groups. G) Quantification of the Masson staining (a) and IHC staining (b,c). Error bars are SEM. *p<0.05, ^**^ *p*<0.01, ^**^ *p*<0.001, and ^****^ *p*<0.0001 by two‐way ANOVA followed by the Sidak post–hoc test. Red asterisk, versus AAV‐NC‐sham group. Blue asterisk, versus AAV‐FBLN7‐sham group.

To further evaluate whether FBLN7 overexpression influences post‐MI cardiac remodeling in vivo, adeno‐associated virus serotype 9 (AAV9)‐mediated gene delivery was used to overexpress FBLN7 in mouse hearts. The efficiency of FBLN7‐overexpression was confirmed by RT‐PCR (Figure S2E, Supporting Information), ELISA (Figure S2F, Supporting Information), and IF (Figure S2G, Supporting Information). The results of echocardiography and cardiac H&E staining were consistent with the previously described results, demonstrating the success of the modeling (**Figure** **3**A,B).

Overexpression of FBLN7 resulted in exacerbated adverse post‐MI remodeling with reduced cardiac function and dilated LV compared with MI mice injected with negative control (AAV‐NC) (Figure 3A–C). Masson and Sirius red staining showed that FBLN7 overexpression aggravated myocardial fibrosis and collagen deposition in the border and remote areas of the infarcted hearts compared to the control group (Figure 3F–H, panel a). Consistently, as shown by IHC staining (Figure 3F,G, panel b,c), excessive deposition of collagen I and III in the non‐infarct area of post‐MI hearts was aggravated by FBLN7 overexpression. Although FBLN7 overexpression also led to a more critical deposition of collagen in the infarct area, the difference in collagen I and III levels in the infarcted zone between FBLN7‐overexpressing mice and AAV‐NC‐injected mice did not reach statistical significance. WB analysis revealed that the levels of collagens I and III were significantly higher in FBLN7‐overexpressing mice (AAV‐FBLN7‐MI) than in AAV‐NC‐MI mice after MI (Figure 3D,E). Taken together, FBLN7 overexpression aggravated myocardial fibrosis and cardiac dysfunction after MI.

### 2.5 FBLN7‐Mediated Post‐MI Cardiac Fibrosis is Associated with Activated Fibroblasts

**Figure 4:**
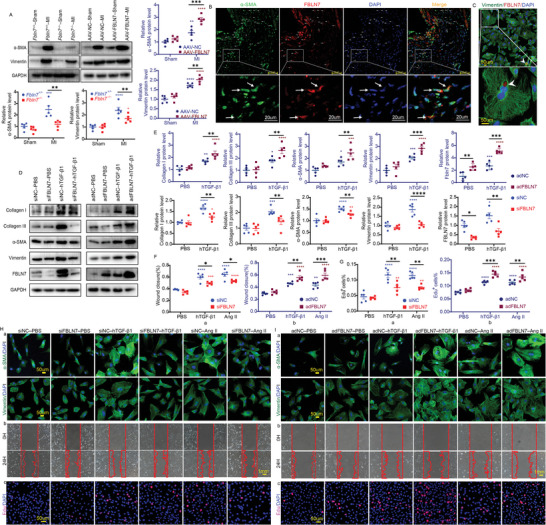
FBLN7 modulates cardiac fibroblast activation in vivo and in vitro. A) Representative western blot images of alpha‐smooth muscle actin (*α*‐SMA) and vimentin proteins of heart tissues from FBLN7 deletion (Fbln7^−/−^), FBLN7 overexpression (AAV‐FBLN7) and corresponding controls (Fbln7^+/+^ and AAV‐NC) mice, after myocardial infarction (MI) or sham operation., Quantifications were shown at the right and bottom (*n* = 5 per group). B) Representative immunofluorescent (IF) micrographs of co‐localization of FBLN7 and *α*‐SMA in the wild‐type infarcted heart. Arrows indicate representative co‐localization. C) A representative IF picture of FBLN7 and vimentin co‐stained cardiac fibroblasts (CFs) isolated from infarcted hearts of wild‐type mouse. Arrows indicate a representative vesicle transporting FBLN7. D–I) Effects of FBLN7 overexpression (adFBLN7) and silencing on recombinant human TGF‐*β*1 (hTGF‐*β*1) or Ang II‐induced fibroblast activation in vitro. D) Representative western blot images of collagen I and III, *α*‐SMA, vimentin, and FBLN7 proteins in FBLN7 silencing and overexpression CFs after treatment with hTGF‐*β*1 or PBS. Quantifications are shown in (E). H) FBLN7 silencing inhibited the migration (b), proliferation (c), and transdifferentiation into myofibroblasts (a) of hTGF‐*β*1 or Ang II‐stimulated CFs. (a) Representative IF images of CFs stained with *α*‐SMA (green) and vimentin (green), respectively, with nuclei stained with DAPI (blue). (b) Representative photomicrographs of scratch wounds at 0 and 24 h after wounds were made. Red lines mark the scratch edges. Quantification of migration distances is shown in (F, panel a). (c) Representative images of CFs co‐stained with EdU (red) and DAPI (blue). Quantification of percentage EdU‐positive cells shown in (G, panel a). I) FBLN7 overexpression promoted the migration (b), proliferation (c), and transdifferentiation into myofibroblasts (a) of hTGF‐*β*1 or Ang II‐stimulated CFs. (a) Representative IF images of CFs stained with *α*‐SMA (green) and vimentin (green), respectively. (b) Representative images of scratch wounds at 0 and 24 h after wounds were made. Quantification is shown in (F, panel b). (c) Representative images of CFs co‐stained with EdU (red) and DAPI (blue). Quantification is shown in (G, panel b). Error bars are SEM. *p<0.05, ^**^ *p*<0.01, ^***^ *p*<0.001, and ^****^ *p*<0.0001 by Two‐way ANOVA followed by Sidak post hoc test. Red and blue asterisks, compared with corresponding sham‐operated or PBS‐treated groups.

Given the critical role of myofibroblasts (also termed activated fibroblasts) in post‐MI myocardium fibrosis, we investigated the effect of FBLN7 on the activation of cardiac fibroblasts (CFs). The non‐infarcted myocardium from MI mice and the myocardium from sham mice were collected for western blotting. The results showed that FBLN7 deletion reduced the upregulation of myofibroblast‐associated alpha‐smooth muscle actin (*α*‐SMA) and vimentin induced by MI, while FBLN7 overexpression further promoted the upregulation of these proteins (**Figure** **4**A). Immunofluorescence (IF) staining of *α*‐SMA and vimentin revealed similar results (Figure 3A,B, Supporting Information). Moreover, the overexpression of FBLN7 induced an increase in *α*‐SMA and vimentin protein levels in AAV‐FBLN7‐sham mice as compared to AAV‐NC‐sham mice, though this effect did not reach statistical significance (Figure 4A). The regulation of myofibroblast marker expression by FBLN7 indicates that FBLN7 is involved in the transformation of fibroblasts into myofibroblasts. Meanwhile, the confocal imaging revealed a co‐localization of myofibroblasts (labeled with *α*‐SMA) and FBLN7 in the infarcted hearts (Figure 4B). Immunocytochemistry (ICC) staining of CFs isolated from infarcted hearts revealed the distribution of FBLN7‐positive transport vesicles in vimentin‐labeled CFs (Figure 4C). Collectively, these results demonstrate that FBLN7 may mediate post‐MI cardiac fibrosis by activating CFs.

### 2.6 FBLN7 Modulates Cardiac Fibroblast Activation In Vitro

CFs from neonatal mice were cultured and separately incubated with recombinant human transforming growth factor‐*β*1 (hTGF‐*β*1) and angiotensin II (Ang II) to mimic the fundamental biological process leading to fibrotic diseases in vitro. After hTGF‐*β*1 or Ang II stimulation, the levels of FBLN7, myofibroblast markers *α*‐SMA and vimentin, and fibrotic markers collagen I and III were significantly elevated in CFs treated with control adenovirus (adNC) or control siRNA (siNC) (Figure 4D,E). ICC staining of *α*‐SMA and vimentin, scratch migration assay, and 5–ethynyl–2'–deoxyuridine (EdU) assay also demonstrated enhanced fibroblast migration, proliferation, and trans‐differentiation into myofibroblasts after hTGF‐*β*1 or Ang II stimulation (Figure 4F–I), indicating the successful construction of in vitro fibrotic models. CFs were then transfected with an FBLN7‐targeted siRNA (siFBLN7) or with an adenovirus expressing FBLN7 (adFBLN7) to further examine the effects of FBLN7 silencing and overexpression on fibroblast activation. FBLN7 overexpression enhanced the hTGF‐*β*1‐induced fibroblast migration (Figure 4I,F, panel b), proliferation (Figure 4I,G, panel c), and trans‐differentiation into myofibroblasts (Figure 4D,E,I, panel a); in contrast, FBLN7 silencing effectively inhibited the fibroblast activation induced by hTGF‐*β*1(Figure 4D–H). The renin‐angiotensin system (RAS) is activated during post‐MI remodeling; therefore, the promotion of fibroblast activation by FBLN7 was further verified in Ang II‐induced hypertrophic responses. Ang II‐treated CFs with FBLN7 overexpression exhibited further increased *α*‐SMA and vimentin levels (Figure S4, Supporting Information; Figure 4I, panel a), collagen synthesis (collagen I and III) (Figure S4, Supporting Information), migration (Figure 4F,I), and proliferation (Figure 4I,G, panel c) compared with Ang II‐stimulated control CFs; in contrast, FBLN7 knockdown elicited an attenuated Ang II‐induced fibroblast activation (Figure S4, Supporting Information; Figure 4F–H). Notably, overexpression of FBLN7 alone triggered the activation of CFs, and FBLN7 downregulation alone resulted in a mild inhibition of fibroblast activation, although these trends did not reach statistical significance. These data indicated that FBLN7 is involved in the regulation of hTGF‐*β*1 or Ang II‐induced CF activation and the production of collagens.

### 2.7 FBLN7 Regulates Fibroblast Activation via the FAK/AKT Pathway

**Figure 5:**
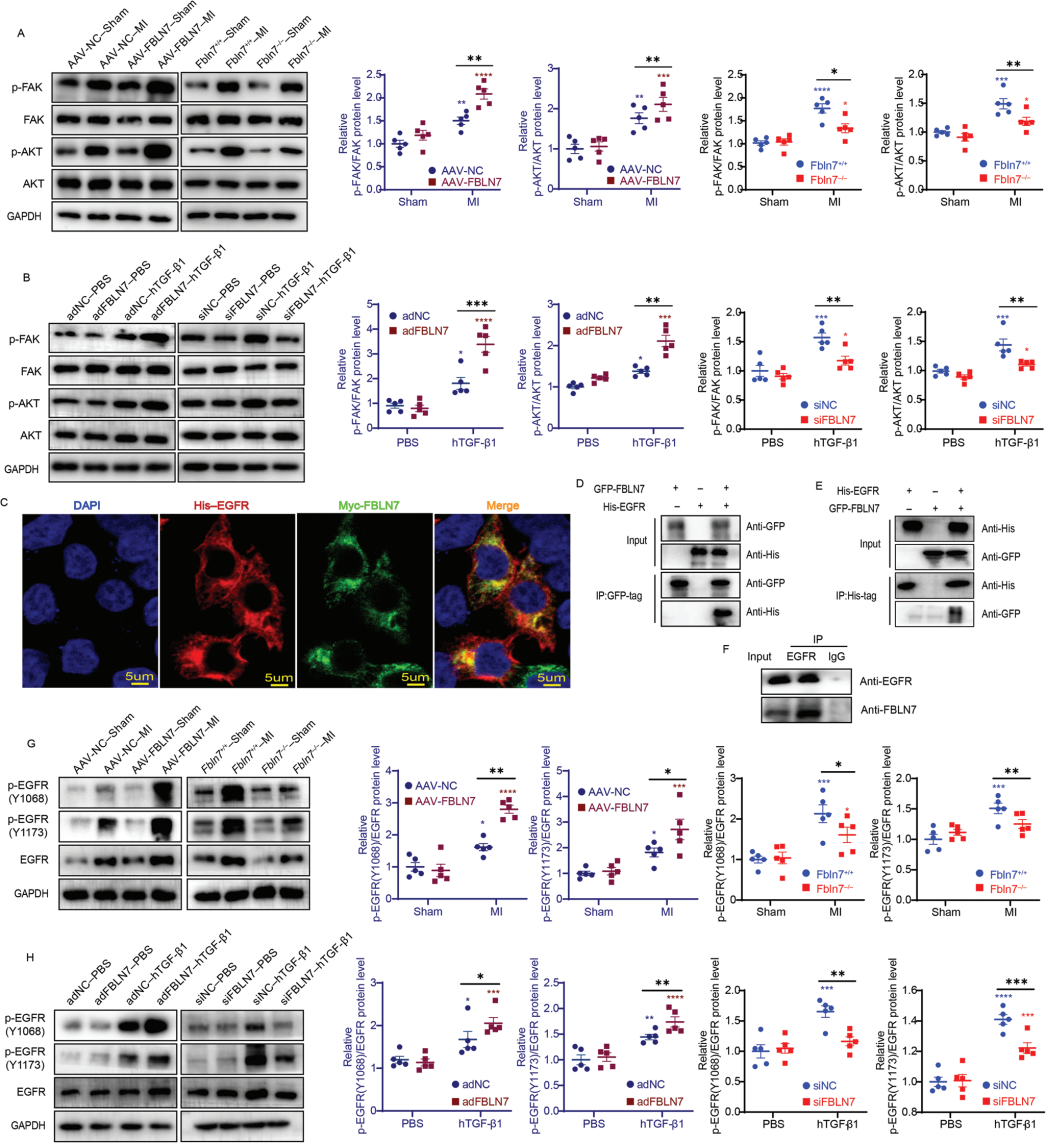
FBLN7 modulates EGFR autophosphorylation and the activation of intracellular FAK/AKT signaling. A) Representative western blot images of p‐FAK, FAK, p‐AKT, and AKT proteins in heart tissues from FBLN7 deletion (Fbln7^−/−^), FBLN7 overexpression (AAV‐FBLN7) and corresponding control mice following MI or sham operation. Quantifications of the band intensity were shown on the right. B) Representative western blot images of p‐FAK, FAK, p‐AKT and AKT levels in FBLN7 silencing (siFBLN7), FBLN7 overexpression (adFBLN7) and corresponding control cardiac fibroblasts (CFs) after treatment with hTGF‐*β*1 or PBS. C) Representative confocal images of co‐localization of Myc‐FBLN7 and His‐EGFR in 293T cells. D–F) Co‐IP of FBLN7 and EGFR. D) IP with green fluorescent protein (GFP)‐FBLN7 and IB with His‐EGFR in 293T cells. E) IP with His‐EGFR and IB with GFP‐FBLN7 in 293T cells. F) IP with EGFR and IB with FBLN7 in hTGF‐*β*1‐treated CFs. G,H) Representative western blot images of p‐EGFR(Y1068), p‐EGFR(Y1173), and EGFR protein levels in (G) heart tissues from FBLN7 KO and overexpression mice following MI or sham operation, and in (H) FBLN7 silencing and overexpression CFs after treatment with hTGF‐*β*1 or PBS. Error bars are SEM. *p<0.05, ^**^ *p*<0.01, ^***^ *p*<0.001, and ^****^ *p*<0.0001 by two‐way ANOVA followed by the Sidak post–hoc test. Red and blue asterisks, versus corresponding sham or PBS group.

The focal adhesion kinase (FAK) is a key regulator of the focal adhesion pathway, which was evidently enriched in the KEGG analysis of DEGs described previously, and its signal cascade, the FAK/AKT pathway, is involved in the development of cardiac hypertrophy by regulating various cellular responses.^[^ ^31^, ^32^ ^]^ We then investigated whether the FAK/AKT pathway is involved in FBLN7‐driven fibroblast activation during cardiac remodeling after MI. In vivo, MI increased phospho‐FAK and phospho‐AKT levels without altering total FAK or AKT levels. Compared with the AAV‐NC‐MI group, the phosphorylation levels of FAK and AKT were further increased in the AAV‐FBLN7‐MI group, while the phosphorylation levels of FAK and AKT were significantly decreased in the Fbln7^−/−^‐MI group compared with the Fbln7^+/+^‐MI group (**Figure** **5**A). In vitro, the phosphorylation levels of FAK and AKT were also upregulated in CFs stimulated with hTGF‐*β*1 (Figure 5B). FBLN7 overexpression led to a further increase in phosphorylation levels of FAK and AKT in the adFBLN7‐hTGF‐*β*1 group as compared to the adNC‐hTGF‐*β*1 group, and great decreases in p‐FAK/FAK and p‐AKT/AKT ratios were displayed in the siFBLN7‐hTGF‐*β*1 group relative to the siNC‐hTGF‐*β*1 group (Figure 5B). Although the effect of FBLN7 silencing or overexpression on the FAK/AKT signaling pathway was in line with the effect on fibroblast activation, the association between FBLN7 and FAK in lysates from CFs stimulated with hTGF‐*β*1 was not detected by co‐immunoprecipitation (Co‐IP) (Figure S5, Supporting Information). These findings demonstrated that the FAK/AKT pathway is involved in the regulation of FBLN7 on MI/hTGF‐*β*1‐induced fibroblast activation, though FAK may not be a direct target of FBLN7.

### 2.8 FBLN7 Binds to EGFR and Activates its Kinase Activity

Epidermal growth factor receptor (EGFR) is a crucial membrane tyrosine kinase. It serves as a critical component of the ECMreceptor pathway and is involved in the pathological process of cardiac remodeling.^[^ ^33^ ^]^ Its activation initiates various downstream signaling cascades, including the FAK and AKT pathways. Critically, FBLN7 may interact with EGFR,^[^ ^34^ ^]^ suggesting that FBLN7‐promoted fibroblast activation involves interaction with EGFR. First, confocal images showed the co‐localization of exogenous His‐EGFR and Myc‐FBLN7 in 293T cells (Figure 5C), and co‐IP experiments revealed that exogenously expressed His‐tagged EGFR co‐precipitated with the co‐expressed green fluorescent protein (GFP)‐tagged FBLN7 in 293T cells (Figure 5D), and vice versa (Figure 5E). Co‐IP of endogenous EGFR and FBLN7 in CFs also confirmed this interaction (Figure 5F), indicating that FBLN7 binds to EGFR. We then explored in vivo and in vitro whether the FBLN7‐EGFR interaction triggered the phosphorylation of EGFR at tyrosine (Y) 1068 and Y1173, the major sites for EGFR autophosphorylation, indicative of the activation of its tyrosine kinase domain. MI‐induced increases in the levels of p‐EGFR(Y1068)/EGFR and p‐EGFR(Y1173)/EGFR were strikingly enhanced by FBLN7 overexpression but suppressed after FBLN7 knockdown (Figure 5G). The effect of FBLN7 on EGFR autophosphorylation in vitro was consistent with that observed in vivo (Figure 5H). These results collectively indicate that FBLN7 binds to EGFR and increases its autophosphorylation level.

An unexpected observation was that FBLN7 overexpression resulted in a substantial up‐regulation of EGFR protein in heart tissues from MI mice or in hTGF‐*β*1‐stimulated CFs, whereas FBLN7 knockdown resulted in down‐regulation of EGFR protein expression (Figure S6A–C, Supporting Information). However, RT‐PCR demonstrated that FBLN7 deletion or overexpression had no impact on EGFR mRNA levels, suggesting that FBLN7 may regulate EGFR expression post‐transcriptionally.

### 2.9 EGFR Activation is Required for FBLN7‐Mediated Efficient Activity of the FAK/AKT Pathway and Myofibroblast

**Figure 6:**
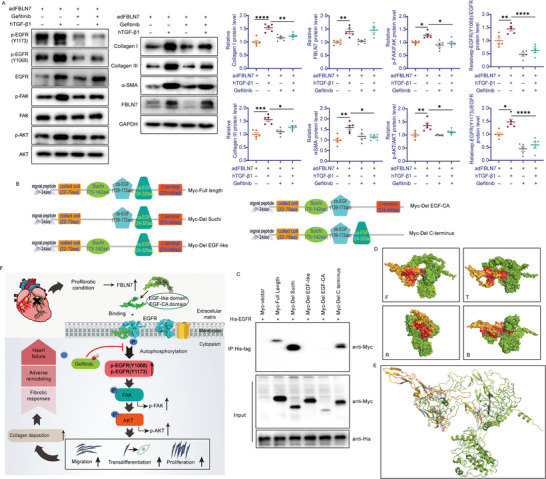
FBLN7 binds with EGFR through its EGF‐like and EGF‐CA domains. A) Representative western blot images showing protein levels of p‐EGFR(Y1068), p‐EGFR(Y1173), EGFR, p‐FAK, FAK, p‐AKT, AKT, collagen I and III, *α*‐SMA, and FBLN7 in cardiac fibroblasts (CFs) infected with adenovirus encoding FBLN7 after treatment with PBS, TGF‐*β*1, or 1 µm gefitinib, or TGF‐*β*1 plus gefitinib. Quantifications are shown on the right. B) Schematic diagram of FBLN7 and its deletion mutants used for EGFR co‐IP experiments in (C). C) Co‐IP assay in 293T cells between His‐EGFR and deletion mutants of Myc‐FBLN7 showing that the EGF‐like domain (136–172 aa) and EGF‐CA domain (224–320 aa) of FBLN7 were both required for binding to EGFR. D) Surface representations and cartoon representation (E) of the FBLN7(136‐320 aa)‐EGFR complex structure model predicted from HADDOCK program in four different views, with FBLN7 polypeptide colored orange, EGFR colored green, and interaction interface colored red. F, front view; T, top view; R, right side view; B, bottom view. F) During the cardiac remodeling following myocardial infarction, the extracellular matrix protein FBLN7 increases and binds with EGFR via its EGF‐like and EGF‐CA domains that promote EGFR autophosphorylation at Y1068 and Y1173, facilitating the phosphorylation of downstream FAK and AKT, which contributes to CF proliferation, migration and trans‐differentiation to myofibroblast and ultimately leads to excessive collagen deposition and cardiac fibrosis. These FBLN7‐EGFR interaction‐mediated signal transductions and fibrotic responses could be effectively inhibited by Gefitinib. Error bars are SEM. *p<0.05, ^**^ *p*<0.01, ^***^ *p*<0.001, and ^****^ *p*<0.0001 by one‐way ANOVA followed by the Tukey post–hoc test.

Since FBLN7 promotes fibroblast activation by activating FAK/AKT signaling, which is downstream of EGFR,^[^ ^35^ ^]^ it is likely that FBLN7 modulates FAK/AKT signaling by regulating EGFR. Gefitinib was used to inhibit EGFR tyrosine kinase activity in CFs. The p‐EGFR(Y1068)/EGFR and p‐EGFR(Y1173)/EGFR ratios dramatically decreased in the presence of gefitinib. The promotion effect of FBLN7 overexpression on fibrotic responses and FAK/AKT pathway activation in hTGF‐*β*1‐treated CFs was largely abrogated with the inhibition of EGFR autophosphorylation, compared with hTGF‐*β*1‐treated CFs overexpressing FBLN7 alone, as evidenced by decreased p‐FAK/FAK and p‐AKT/AKT, *α*‐SMA, collagen I, and collagen III protein levels (**Figure** **6**A). Moreover, the inhibitory effect of FBLN7 knockdown on hTGF‐*β*1‐induced FAK/AKT pathway activation and fibrotic responses in the CFs was partially counteracted after treatment with an EGFR agonist (NSC 228155) (Figure S6D, Supporting Information). These findings indicate that FBLN7 promotes hTGF‐*β*1‐induced FAK/AKT signaling activation and subsequent fibroblast activation, which are largely dependent on EGFR kinase activity.

### 2.10 FBLN7 Binds to EGFR through the EGF‐Like and EGF‐Like Calcium‐binding Domains

To explore the key domains of FBLN7 that are required for EGFR binding, the primary structure of the FBLN7 protein was analyzed, and four predicted functional domains were identified: the sushi domain (sushi, residues 73‐142), EGF‐like domain (EGF‐like, residues 136‐172), EGF‐like calcium‐binding domain (EGF‐CA, residues 224‐320, consisting of two EGF‐like domains), and C‐terminus (residues 374‐440) (Figure 6B). Full‐length FBLN7 and four truncated fragments were expressed as Myc fusion proteins. Western blotting of the immunoprecipitated fraction of the full‐length His‐EGFR showed that FBLN7 lacking EGF‐like or EGF‐CA lost the ability to bind to EGFR, whereas full‐length FBLN7, mutants lacking sushi, and mutants lacking the C‐terminus equally bound to EGFR (Figure 6C). In addition, a simple molecular modeling study was performed to provide a structure‐based rationalization for the interaction between the EGF‐like and EGF‐CA domains of FBLN7 and EGFR. The high ambiguity‐driven protein–protein docking (HADDOCK) program clustered 59 water‐refined structures into seven clusters. Models in cluster 6 were the most reliable based on the HADDOCK score (88.6 +/− 28.9) and Z‐score (−1.9). The best model selected according to protein, interface, structure, and assembly (PISA) analysis showed that the EGF‐like and EGF‐CA domains of FBLN7 interacted with the extracellular domains I (L1) and III (L2) of EGFR with a buried surface area of 3276 Å2 (Figure 6D). The interaction interface of this complex involved 28 hydrogen bonds (distances ≤3.5 Å), six salt bridges, and extensive hydrophobic interactions, indicating a strong interaction between the FBLN7 and EGFR proteins (Figure 6E,F). The residues involved in the polar interactions are listed in Table S2 (Supporting Information). These data indicate that the EGF‐like and EGF‐CA domains of FBLN7 are responsible for binding to EGFR.

## 3 Discussion

Fibulins are an important class of matrix proteins that influence a variety of cell processes as their complex interactions with other ECM molecules and cell receptors.^[^ ^10^, ^36^ ^]^ Several fibulins, including FBLN2,^[^ ^37^ ^]^ fibulin 6,^[^ ^38^ ^]^ and FBLN3^[^ ^39^ ^]^ have been reported to participate in the regulation of cardiac fibrosis. It is possible that FBLN7 actively participates in fibrosis. However, experimental evidence is lacking.

In this study, we determined that FBLN7 deletion protected the heart against MI‐induced pathological cardiac remodeling, resulting in improved cardiac function and reduced fibrosis, whereas overexpression of FBLN7 had the opposite effects. This is consistent with observations from other studies that fibulin family members are involved in cardiac fibrosis and reinforces the importance of fibulins as matricellular proteins in the development of fibrosis.^[^ ^22^, ^39^, ^40^ ^]^ Therapeutic strategies attenuating cardiac fibrosis may disrupt optimal infarct (scar) formation, leading to post‐MI rupture.^[^ ^41^ ^]^ But the loss of FBLN7 had little effect on post‐MI mortality in the study, suggesting that FBLN7 deletion ameliorated myocardial fibrosis in post‐MI mice without compromising infarct healing. This could be attributed to the FBLN7 peak emerging after the development of the fibrotic scar. Our observations are consistent with those of Tsuda et al., who demonstrated that deficiency of FBLN2, another member of this family, protects against the deposition of collagen I and III and ventricular remodeling after MI without causing additional cardiac rupture.^[^ ^37^ ^]^ In the present study, we found that FBLN7 significantly contributed to diffuse fibrosis in the myocardium remote from the infarct. Given that diffuse fibrosis is commonly seen in chronic heart diseases such as hypertensive heart disease,^[^ ^42^ ^]^ aortic stenosis,^[^ ^43^ ^]^ HCM,^[^ ^44^ ^]^ and some systemic clinical conditions evolving with HF,^[^ ^45^, ^46^ ^]^ this finding raises the possibility that FBLN7 may function in various chronic diseases that can lead to diffuse myocardial fibrosis. However, further studies are required to confirm and validate these hypotheses.

TGF‐*β* is a major profibrogenic cytokine and is a central mediator of fibroblast activation in many tissues. The fibulins have been shown to play certain regulatory roles in TGF‐*β* signaling in various ways.^[^ ^47^ ^]^ Consistent with the prior studies,^[^ ^22^, ^38^, ^40^, ^48^ ^]^ we demonstrated that fibroblasts overexpressing FBLN7 are more susceptible to the profibrotic effects of hTGF‐*β*1 or Ang II, whereas the stimulatory effects of hTGF‐*β*1 or Ang II on myofibroblast differentiation and collagen release are reduced in FBLN7 knockdown fibroblasts. Myofibroblasts are the central cellular effectors in cardiac fibrosis and serve as the primary source of matricellular proteins.^[^ ^49^ ^]^ In this study, we found co‐localization of FBLN7 with myofibroblasts in the infarcted heart and the presence of FBLN7 in vesicles transported by CFs isolated from infarcted hearts, which further highlights the interaction between FBLN7 and myofibroblasts. In addition, previous studies indicated that matricellular proteins indirectly modulate fibroblast function (through actions on immune and vascular cells that may modulate fibroblast activation).^[^ ^5^ ^]^ It is possible that FBLN7 modulates fibroblast activation by first interacting with macrophages, as we observed the co‐localization of FBLN7 with macrophages in the infarcted heart (data not shown). However, this hypothesis warrants further investigation.

Abnormal EGFR tyrosine kinase activation promotes the development of cardiac fibrosis.^[^ ^33^, ^50^ ^]^ In the present study, we found that EGFR is responsible for transmitting extracellular FBLN7 signals. Our experimental data highlighted that FBLN7 binds to EGFR to activate its kinase function (evidenced by increased autophosphorylation at Y1068 and Y1173) and the subsequent intracellular FAK/AKT signaling pathway, which conveys abnormal EGFR signals from the CF surface to the nucleus, thereby leading to fibrotic responses. Furthermore, the effects of FBLN7 overexpression were largely abrogated in the presence of EGFR inhibitors. Notably, overexpression of FBLN7 increased the protein level of EGFR without affecting its mRNA level in the presence of profibrotic stimuli. The possible reason for this is that increased levels of FBLN7 impaired EGFR internalization, as FBLN7 may participate in vesicle‐related processes including cytoskeleton reorganization and vesicle transport, key events in EGFR internalization.^[^ ^51^, ^52^ ^]^ FBLN7 regulates endothelial cell function through interacting with integrins^[^ ^12^ ^]^ and activated integrins are associated with cytoskeleton reorganization. Rac1 is involved in stabilizing actin cytoskeleton and regulating vesicular trafficking,^[^ ^53^ ^]^ and FBLN7 has been reported to affect its activation.^[^ ^54^ ^]^ FBLN7 may also regulate EGFR internalization by activating FAK, as FAK activation may inhibit actin‐mediated endocytosis.^[^ ^55^ ^]^ Nevertheless, the current study could not prove whether the upregulation of EGFR levels was responsible for the observed activation of EGFR signaling. This deserves further investigation, especially considering their potential roles in tumor progression and resistance to therapy.

The EGF‐like domain is a conserved protein module that is highly homologous to EGF and found in all members of the fibulin family.^[^ ^56^, ^57^ ^]^ The EGF‐like domain mediates interactions between fibulins and ECM proteins,^[^ ^58^, ^59^, ^60^ ^]^ such as the binding ofFBLN1 to fibronectin. Concurrently, EGF‐like domain also plays a key role in activating EGFR, as the known ligands of the EGF family, including EGF, TGF‐alpha, amphiregulin, betacellulin, epiregulin, and heparin‐binding EGF, all contain EGF‐like domains.^[^ ^61^ ^]^ Similarly, EGF‐like domains were responsible for the binding of FBLN7 to EGFR in this study. Hou et al. previously reported that recombinant partial FBLN7 (containing an EGF‐CA domain) could interact with adhesin‐like protein (ALP) and that recombinant ALP inhibit EGF‐induced EGFR activation.^[^ ^58^ ^]^ Taken together, these findings suggest that the inhibition of EGFR by recombinant ALP is a result of competition with EGFR for binding to the EGF‐CA domain of FBLN7. This not only further supports our results but also helps guide the identification of potential targets to inhibit FBLN7 in the future.

Although these results support our conclusion, further investigation is needed to determine whether FBLN7 may also contribute to the phenotype switching of other heart cell types because it is a secreted protein and whether FBLN7 from other heart cell types or organs may promote post‐MI cardiac fibrosis since it is present in the circulating blood. Our study is based on a global FBLN7 deletion.

In conclusion, we discovered a profibrotic role of FBLN7 in post‐MI cardiac fibrosis, which may be mediated by the activation of the EGFR/FAK/AKT pathway to promote fibroblast activation.

## 4 Experimental Section

### Differential Expression Analysis

The microarray dataset was obtained from a publicly available gene expression dataset (GSE1145). The dataset contained raw data from microarrays performed on left ventricular free wall tissue. Tissues were obtained from heart failure samples at the time of cardiac surgery after transplantation. The empirical Bayes method in the R software package LIMMA (version 3.40.6) was used to compute the differential expression of genes in the microarray data analysis. The p‐values determined using the *t*‐test were then subjected to multiple test corrections using the false discovery rate method to correct false‐positive occurrences and obtain statistically significant genes. The minimum absolute value of log (fold change) 1.2 was determined as the threshold cutoff value for the DEGs. A volcano plot was constructed on the DEGs, and heat maps were generated on the normalized expression profiles of the top 50 variable genes with significantly adjusted p‐values. Genes were based on Euclidean distance.

### Gene Enrichment Analysis

For the functional enrichment analysis of gene sets, GO annotations of genes in the R software package org.hs.eg.db (version 3.1.0) and KEGG pathway gene annotations obtained from the KEGG rest API were used as background sets. DEGs were then mapped to background sets. The R software package Cluster Profiler (version 3.14.3) was used for the enrichment analysis. Statistical significance was set at *p* < 0.05.

### Study Population and Blood Samples

Thirty‐nine patients with adverse cardiac remodeling after MI and 37 without MI were recruited from Shandong University Affiliated Qilu Hospital. Cardiac remodeling was defined as the presence of a left ventricular ejection fraction (LVEF) ≤ 50%^[^ ^62^ ^]^ and/or a left ventricular internal diameter at diastole (LVIDD) ≥ 55 mm.^[^ ^63^ ^]^ This study was approved by the Institutional Clinical Research and Ethics Committee. All patients and their representatives provided written informed consent. This study complied with the principles of the Declaration of Helsinki.

### Animals

The generation of the Fbln7‐knockout mice (Fbln7^−/−^) was commissioned by Cyagen Biosciences Inc. (Guangzhou, China). To generate knockout mice, the CRISPR/Cas9 strategy was used to delete exons 2–3 of mouse Fbln7 via non‐homologous recombination. Fbln7^−/−^ mice were then obtained by mating the Fbln^+/−^ mice. Subsequent genotyping was performed by PCR using the genomic DNA obtained from clipped tails with the following primers: forward 5’‐AAGACATAAACATCAACCTCTGGC‐3’ and reverse 5’‐CACATTGCTCTTGCATTTGTGTG‐3.’

The adeno‐associated virus serotype 9 (AAV9) vector with the cytomegalovirus (CMV) promoter containing the gene sequence for FBLN7 (AAV‐FBLN7) was used for in vivo overexpression. An AAV9‐empty vector (AAV‐NC) was used as a control (GeneChem, Shanghai, China).

C57BL/6 mice (6–7 weeks old) were randomly injected with AAV‐FBLN 7 or AAV‐NC via the tail vein at 2.5 × 10^11^ vector genomes (vg) per mouse.

### Animal Model

Age‐matched male C57BL/6 mice (8–12 weeks old) were used as an MI mouse model to simulate cardiac fibrosis, ventricular remodeling, and heart failure. As previously described, left anterior descending artery (LAD) ligation was performed to induce MI.^[^ ^64^ ^]^ Sham‐operated mice underwent a similar procedure, except for LAD ligation. The mice were randomly divided into MI and sham groups. At least five mice were included per group for each experiment. All mice experiments were carried on following the general guidelines published by the Association for Assessment and Accreditation of Laboratory Animal Care, approved by the Laboratory Animal Committee of Shandong University Qilu Hospital (Jinan, Shandong Province, China) (Permit number: DWLL–2021–165).

An ISO‐induced mouse model of cardiac hypertrophy was established by subcutaneous injection of isoproterenol hydrochloride (ISO, 60 mg per kg per day, diluted in sterile normal saline (NS), Sigma–Aldrich Corporation, St. Louis, MO, USA) once per day for 14 days. The control mice were injected with equal volumes of sterile NS.

### Western Blot

Cells and heart tissues were collected, and lysates were prepared. An equal quantity of protein (40–60 µg) was resolved by SDS/PAGE and transferred to polyvinylidene difluoride membranes. The blots were blocked with 5% milk in Tris‐buffered saline with Tween 20 for 2 h at room temperature (RT) and then separately incubated with primary antibodies overnight at 4°C. The blots were then incubated with secondary antibodies conjugated to horseradish peroxidase for 1 h at RT and detected using a chemiluminescent instrument (GE, Amersham Imager 680RGB). Grayscale values for each blot were measured using the ImageJ software (National Institutes of Health, Bethesda, MD, USA), and the intensity of each band was normalized to that of the loading control GAPDH or the total target protein. Detailed information regarding the antibodies used is provided in Table S3 (Supporting Information).

### Quantitative Real‐Time Reverse Transcriptase Polymerase Chain Reaction

Total RNA was extracted using TRIzol reagent (Invitrogen), and the quality was tested. The extracted RNA was reverse transcribed into cDNA using the PrimeScript RT reagent kit with gDNA Eraser (RR047A, TaKaRa, Japan), and then quantified by real‐time polymerase chain reaction (PCR) using SYBR Green PCR Master Mix (RR420A, Takara, Japan) on a CFX96 Real‐time PCR Detection System (Bio‐Rad) following the manufacturer's protocol. Data were normalized to GAPDH expression levels. PCR conditions were: 95 ˚C for 30s followed by 40 cycles of [95 ˚C for 5 s, 55 ˚C for 30 s, and 72 ˚C for 30 s]. The PCR primers used were as follows: Fbln7 forward [GAAGACATCTCCCTTTCAGTGCG] and Fbln7 reverse [GGCATCC].

TCAGAAGTCATAGCG], Gapdh forward [AGGTCGGTGTGAACGGATTTG], Gapdh reverse [TGTAGACCATGTAGTTGAGGTCA].

### Enzyme‐linked immunosorbent assay

Serum FBLN7 levels in patients and mice were determined using enzyme‐linked immunosorbent assay (ELISA) kits following the manufacturer's instructions (Mmbio, Jiangsu, China).

### Echocardiography

Transthoracic echocardiography using a Visual Sonics Vevo 2100 system with an MS‐400 linear transducer (Visual Sonics Vevo 2100) was used to measure the parameters of ventricular remodeling and cardiac function, including LVIDD, left ventricular internal diameter at systole (LVIDS), LVEF, and percent fractional shortening (FS%). The procedure was performed before and 28 days after surgery. Five consecutive cardiac cycles were observed, and the results were counted.

### Histopathological Staining

Isolated heart tissues were immediately fixed in a 4% paraformaldehyde solution, followed by routine dehydration, paraffin embedding, and serial sectioning (4 µm).

- H&E staining was conducted according to standard protocols.
- Masson's staining was performed using a modified Masson's trichrome staining kit (G1346, Solarbio, Beijing, China,).
- Picrosirius red staining was performed by staining the heart sections in a freshly prepared 0.1% picrosirius red solution for 2 h at room temperature in the dark.
- For IHC staining, the heart sections were deparaffinized in xylene and rehydrated in graded ethanol. Antigen retrieval was performed according to the methods recommended in the Antibody Manual. Endogenous peroxidase activity was quenched with 3% H2O2 for 15 mins, and sections were blocked in 5% goat serum for 30 mins. Incubations with primary antibodies were performed overnight at 4 °C in a humidified chamber, followed by appropriate HRP‐conjugated secondary antibodies for 1 h at room temperature. A DAB Kit (ZSGB‐Bio, Beijing, China) was used to develop signals according to the manufacturer's instructions. Finally, the sections were counterstained with hematoxylin.
- Immunofluorescence (IF) staining of heart slides was performed similarly to IHC staining, except that the sections were blocked in 5% goat serum after antigen retrieval and subsequently incubated with primary antibodies overnight. The next day, the slides were incubated with Alexa Fluor‐conjugated secondary antibodies for 1 h at room temperature and mounted using a mounting medium containing DAPI (Abcam, ab104139).

The antibodies used are detailed in Table S3 (Supporting Information).

Confocal images were captured using a confocal laser scanning microscope (Carl Zeiss, Göttingen, Germany). Other stained sections were visualized and photographed using the Pannoramic scanner with Pannoramic viewer software (3D HISTECH, Budapest, Hungary) and analyzed with Image‐Pro Plus 6.0 (Media Cybernetics, Sarasota, FL, United States).

### Cardiac Fibroblast Isolation and Culture

Cardiac fibroblasts (CFs) were isolated from neonatal mice, as previously described.^[^ ^65^ ^]^ Neonatal mouse CFs were cultured in DMEM (Basal Media, Shanghai, China) with 10% fetal bovine serum (FBS) (Biological Industries, Israel) and 1% penicillin/streptomycin at 37°C under 5% CO2 in a humidified incubator.

### Cardiac Fibroblast Treatment

Myofibroblast differentiation was induced for 24 h using 10 ng ml^−1^ recombinant human TGF‐*β*1(hTGF‐*β*1, R&D Systems, Germany) or 0.1 µm Angiotensin II (Ang II, Sigma–Aldrich, USA). An equal volume of PBS was used as the control.

When indicated, CFs were pretreated with 1 µmol L^−1^ gefitinib (MCE, Shanghai, China), 0.8 µmol L^−1^ NSC 228155 (Selleck, USA), or 0.01% DMSO (Sigma–Aldrich) for 1 h prior to 24‐hour treatment with hTGF‐*β*1 or Ang II.

### Constructions of Plasmid, siRNA, and Adenovirus

Full‐length cDNA of mouse EGFR and FBLN7 were amplified by standard PCR and subcloned into the pcDNA 3.1 vector with a C‐terminal His‐tag and a C‐terminal EGFP‐tag. Expression plasmids encoding Myc‐tagged full‐length FBLN7 and its truncated mutants were constructed by PCR and cloned into the pcDNA3.1‐Myc vector. pcDNA 3.1‐His, pcDNA 3.1‐Myc, and pcDNA 3.1‐EGFP plasmids were used as controls.

Small interfering RNA (siRNA) oligonucleotides against FBLN7 (siFBLN7) and negative control siRNA (siNC) were synthesized by GenePharma (Shanghai, China).

The sequence of siFBLN7: 5′‐CGUGGUGUGUCUUGCUAAUTT‐3′; siNC: 5′‐UUCUCCGAACGUGUCACGUTT‐3′.

Recombinant adenoviruses encoding FBLN7 (adFBLN7) and an adenovirus vector (adNC served as a negative control) were designed by GeneChem (Shanghai, China).

### Transfection and Infection

CFs were transfected with siFBLN7 and siNC using Lipofectamine RNAiMAX transfection reagent (Invitrogen, USA) to knockdown FBLN7 in vitro, according to the manufacturer's protocol.

For adenovirus‐mediated in vitro overexpression, CFs were incubated with adFBLN7 and adNC for 6–8 h at a multiplicity of infection (MOI) of 50 PFU per cell.

All constructed plasmids were transiently transfected using Lipofectamine 3000 (Invitrogen) according to the manufacturer's instructions.

### Immunocytochemistry (Confocal microscopy)

The cells were fixed in 4% paraformaldehyde for 15 min, permeabilized with 0.1% Triton‐X100 in PBS for 10 min, and blocked in 5% goat serum for 0.5 h at room temperature. Next, the cells were incubated with primary antibodies or PBS (served as a negative control) overnight at 4°C. Appropriate Alexa Fluor‐conjugated secondary antibodies were used for fluorescence staining. Nuclei were stained with DAPI (Abcam, ab104139). Confocal images were acquired using a Zeiss confocal laser scanning microscope (LSM 710, Carl Zeiss, Germany). Other fluorescent immunocytochemical images were captured using a fluorescence microscope (Nikon, Tokyo, Japan).

### Scratch Migration Assay

The scratch migration assay was performed to evaluate the ability of FBLN7 to modulate CF migration. CFs were cultured in 6‐well plates. A straight scratch was made in the middle of each well using a pipette tip when confluency was reached. Next, the medium was replaced with a serum‐free medium. Images of fixed scratch areas were separately captured post‐scratching (0 h) and following 24 hours of incubation at 37°C. Cell migration ability was evaluated based on the migration distance (differences in the widths of scratches before and after migration) measured using ImageJ software 2.0 (National Institutes of Health, Bethesda, MD, USA).

### 5‐Ethynyl‐2′‐Deoxyuridine Assay

The cell proliferation was detected by 5‐Ethynyl‐2′‐Deoxyuridine (EdU) assay using a Cell‐Light EdU DNA Cell Proliferation Kit (RiboBio, Guangzhou, China), in accordance with the manufacturer's protocol. Images were captured using a Nikon fluorescence microscope. The percentage of positive cells was analyzed using Image‐Pro Plus 6.0 (Media Cybernetics, Rockville, MD, USA).

### Co‐Immunoprecipitation

Twenty‐four hours after plasmid transfection, the cells were harvested and lysed in non‐denaturing lysis buffer (P0013, Beyotime Biotechnology). Shanghai, China) for 30 min. Clear lysates were incubated with Immunoprecipitation (IP)‐grade antibodies or IgG for 1 h, followed by the addition of pretreated protein A/G magnetic beads (HY‐K0202; MedChemExpress, Monmouth Junction, NJ, USA) to a rotating wheel at 4°C overnight. The next day, the magnetic beads were recovered, and the supernatant was discarded. After washing the beads four times with PBS containing 0.5% Tween‐20, SDS‐PAGE loading buffer was added, and the mixtures were heated to 95°C for 5 mins. The collected supernatants were used for western blot analysis.

### Molecular Modeling and Docking

The crystal structures of FBLN7 (AF‐Q53RD9‐F1) and EGFR (AF‐P00533‐F1) were obtained from AlphaFold prediction.^[^ ^66^, ^67^ ^]^ A model of the FBLN7 polypeptide (residues 136‐320) was constructed based on the FBLN7 structure. The high ambiguity‐driven protein‐protein docking (HADDOCK) program 2.4 was used to perform the peptide‐protein docking.^[^ ^68^, ^69^ ^]^ The European Bioinformatics Institute (EBI) web service of the Proteins, Interfaces, Structures, and Assemblies (PISA) software was used to perform further interface analysis. All structural figures were prepared using PyMol (PyMOL Molecular Graphics System).

### Statistical Analysis

All results were shown as mean ± SEM unless otherwise indicated. The Kolmogorov–Smirnov test was used to confirm the normal distribution of the data. The unpaired, two‐tailed Student's *t*‐test was used for comparison between two groups with normally distributed data, whereas the two‐tailed Mann–Whitney *U* test was used for data that did not comply with a normal distribution. For univariate comparisons between multiple groups, a one‐way ANOVA followed by Dunnett's or Tukey's post‐hoc tests were used for data with a normal distribution; otherwise, data were analyzed using the Kruskal–Wallis test followed by the Dunn post–hoc test. For multivariate comparisons between groups, a two‐way ANOVA followed by the Sidak post–hoc test was used. GraphPad Prism 8 (GraphPad Software, La Jolla, CA, USA) and IBM SPSS Statistics 25 (IBM Corporation, Armonk, NY, USA) were used for the statistical analyses. Statistical significance was set at *p* < 0.05.

## Conflict of Interest

The authors declare no conflict of interest.

## Author Contributions

X.Z. contributed in conceptualization, investigation, formal analysis, validation, visualization, writing—original draft, review and editing. L.L. contributed in investigation and formal analysis. J.L. contributed in methodology and resources. C.Z. contributed in investigation and validation. J.Z. contributed in conceptualization and supervision. Y.Q. contributed in validation and methodology. L.X. contributed in methodology and resources. C.Z. contributed in validation. G.Y. contributed in methodology and resources. P.B. contributed in conceptualization, resources, writing—review and editing, supervision, project administration, and funding acquisition.

## Supporting information

Supporting Information

## Data Availability

The data that support the findings of this study are available from the corresponding author upon reasonable request.
