# Supplementary material for: Fibulin7 Mediated Pathological Cardiac Remodeling through EGFR Binding and EGFR‐Dependent FAK/AKT Signaling Activation
Source: Adv Sci (Weinh). 2023 Jun 21;10(24):2207631. doi: 10.1002/advs.202207631 (PMC10460860; doi:10.1002/advs.202207631)
Supplement: Supplementary file 1 — Supporting Information [file ADVS-10-2207631-s001.pdf]

ADVANCED SCIENCE
Open Access

Supporting Information

for *Adv. Sci.*, DOI 10.1002/advs.202207631

Fibulin7 Mediated Pathological Cardiac Remodeling through EGFR Binding and EGFR-Dependent FAK/AKT Signaling Activation

*Xuehui Zheng, Lingxin Liu, Jing Liu, Chen Zhang, Jie Zhang, Yan Qi, Lin Xie, Chunmei Zhang, Guoqing Yao and Peili Bu**

Supporting Information

Fibulin7 mediated pathological cardiac remodeling through EGFR binding and EGFR-dependent FAK/AKT signaling activation

*Xuehui Zheng, Lingxin Liu, Jing Liu, Chen Zhang, Jie Zhang, Yan Qi, Lin Xie, Chunmei Zhang, Guoqing Yao, Peili Bu**

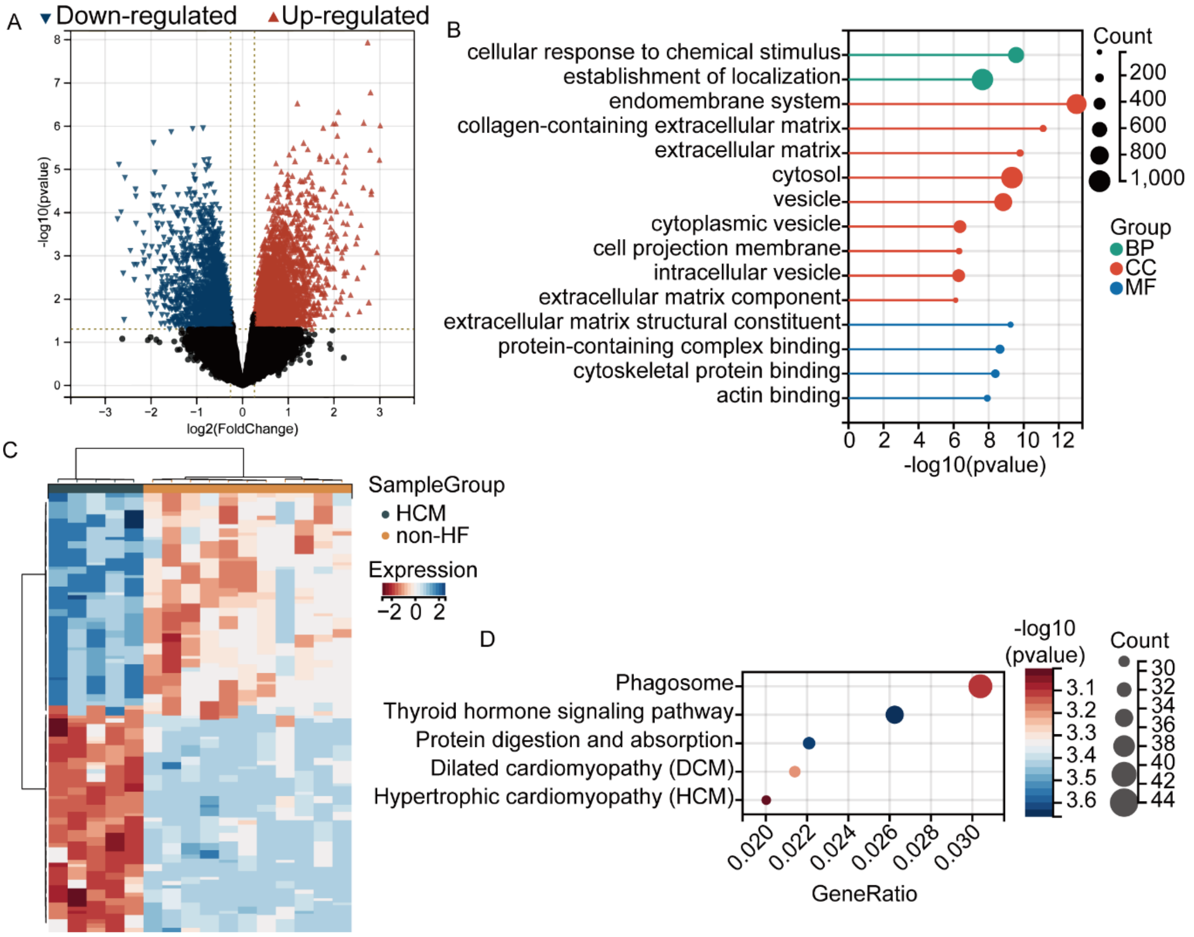

Figure S1. *FBLN7* gene was upregulated in the myocardial samples from hypertrophic cardiomyopathy compared with those from "normal" organ donors. A) Volcano plot of differentially expressed genes (DEGs) between the hypertrophic cardiomyopathy (HCM) (n=5) and "normal" (n=11) samples from the publicly available microarray dataset (GSE1145) from the Gene Expression Omnibus database (|fold change|> 1.2, P<0.05). B) Top 15 enriched gene ontology terms of DEGs (FDR<0.1, P< 0.05). C) Hierarchical clustering heat map showing the top 50 differential genes. Genes are clustered by row, and samples by

column. D) Kyoto Encyclopedia of Genes and Genomes pathway enrichment analysis of DEGs (FDR<0.1, P< 0.05).

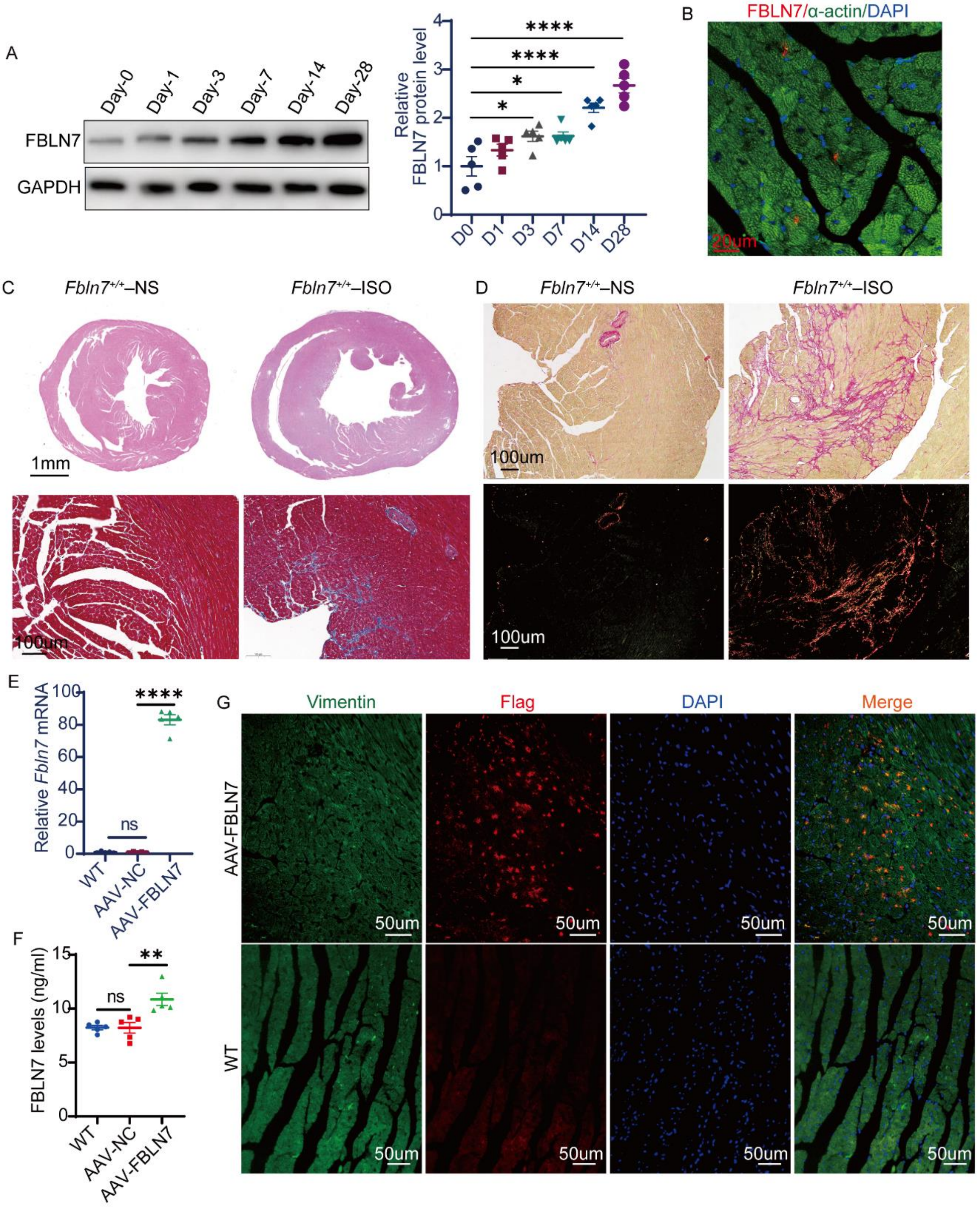

Figure S2. The expression of FBLN7 was gradually increased after myocardial infarction (MI). A) Representative western blot images of FBLN7 protein expression in peri-infarct zones of hearts from wild-type mice at day 1, 3, 7, 14, and 28 post MI. Sham-operated mice were used as control (day 0). B) Representative immunofluorescence staining of FBLN7 and

α-actin in the in the wild-type infarcted heart. C) Representative images of H&E staining and Masson staining of heart sections from mice treated with normal saline (NS) or isoproterenol (ISO). D) Representative images of Sirius Red staining in NS and ISO-treated mice. E-F) Relative mRNA expression level of *Fbln7* and FBLN7 protein levels in myocardium from mice injected with adeno-associated viral (AAV)-FBLN7 and negative control (NC) detected by quantitative real-time PCR (E) and ELISA (F). G) Representative immunofluorescence staining of vimentin and Flag in heart tissues from mice injected with AAV9-FBLN7 or wild-type mice. Error bars are SEM. *P<0.05, **P<0.01, ****P<0.0001, by one-way ANOVA followed by Dunnett post hoc test.

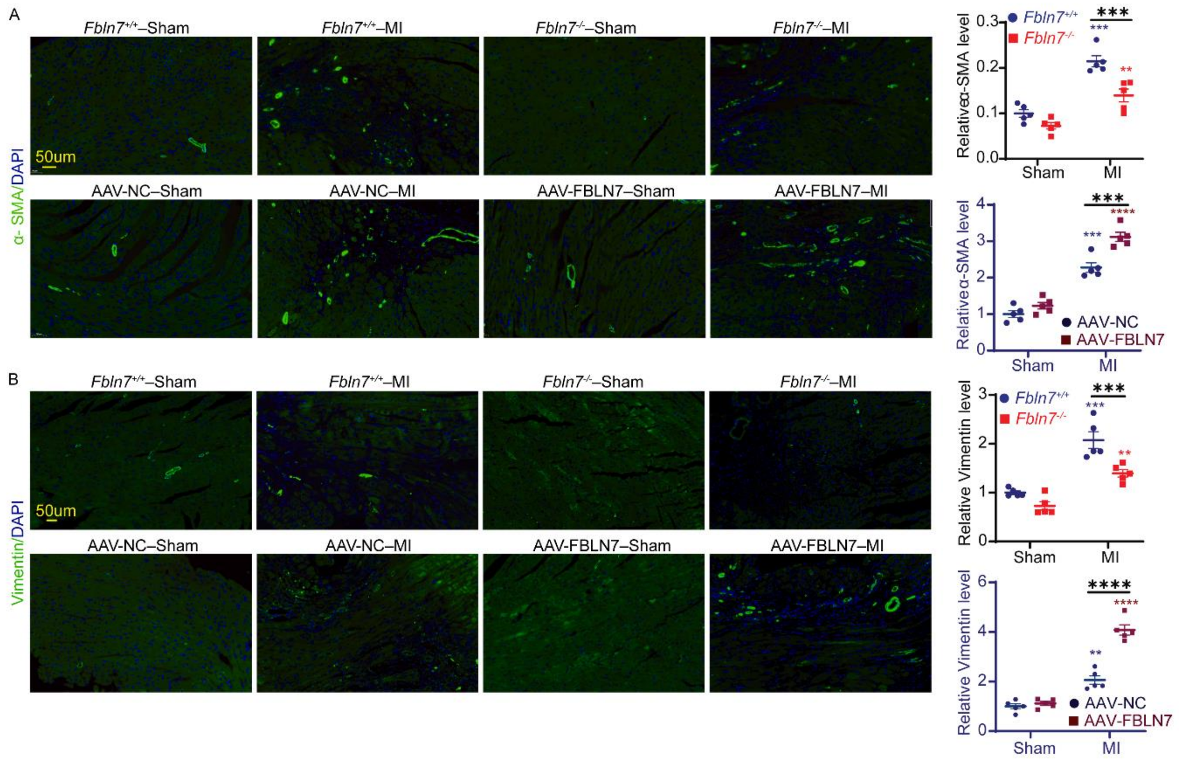

Figure S3. FBLN7 modulates fibroblast activation. A) Representative immunofluorescence (IF) images of alpha smooth muscle actin (α-SMA) and (B) vimentin in heart sections from FBLN7 deletion, FBLN7 overexpression and corresponding control mice, after myocardial infarction (MI) or sham operation. Quantifications were shown on the right. Error bars are SEM. *P<0.05, **P<0.01, ***P<0.001, ****P<0.0001, by two-way ANOVA followed by the Sidak post-hoc test. Red and blue asterisks, versus corresponding sham group.

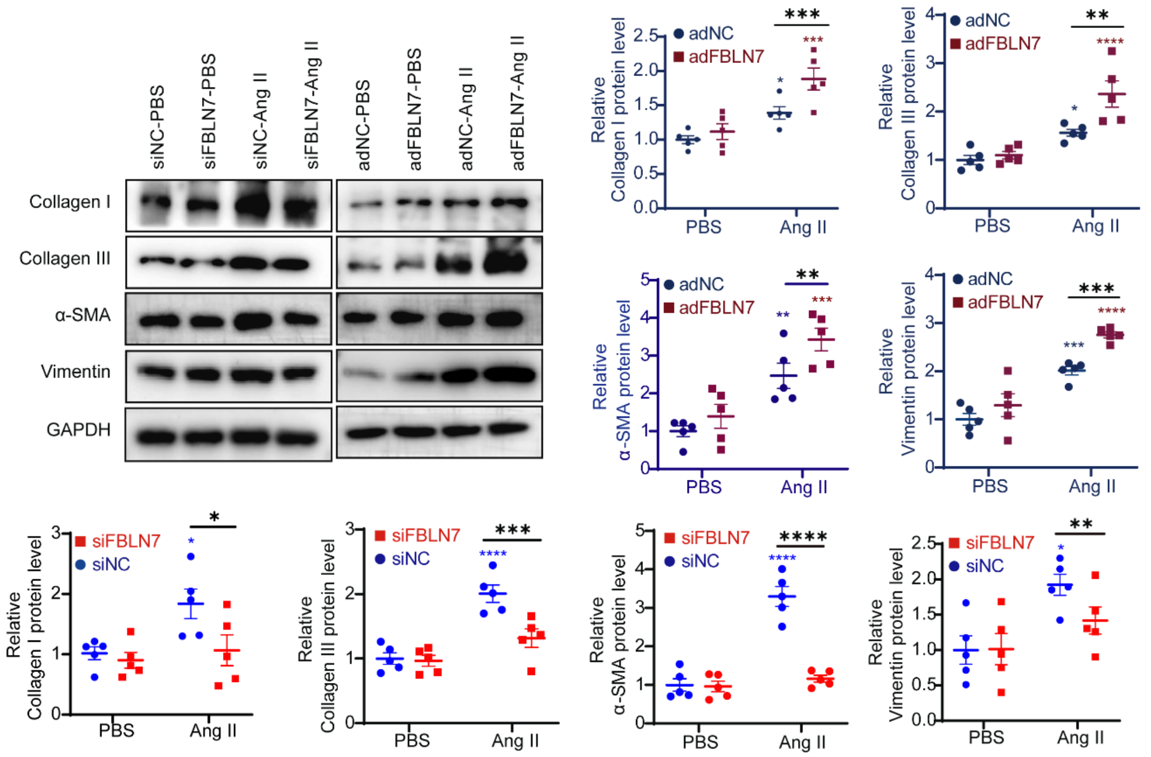

Figure S4. FBLN7 promotes the Ang II-induced fibroblast-to-myofibroblast differentiation. Representative picture and analysis diagram show the protein levels of collagen I and III, α-SMA, vimentin and FBLN7 in Ang II-treated cardiac fibroblasts as determined by western blotting. Protein expression normalized to GAPDH loading control. Error bars are SEM. *P<0.05, **P<0.01, ***P<0.001, ****P<0.0001 by two-way ANOVA followed by the Sidak post-hoc test.

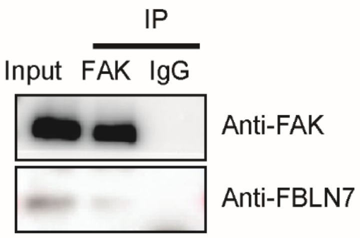

Figure S5. Co-IP of FBLN7 and FAK. IP using anti-FAK antibody and IB using anti-FBLN7 antibody in hTGF-β1–stimulated cardiac fibroblasts.

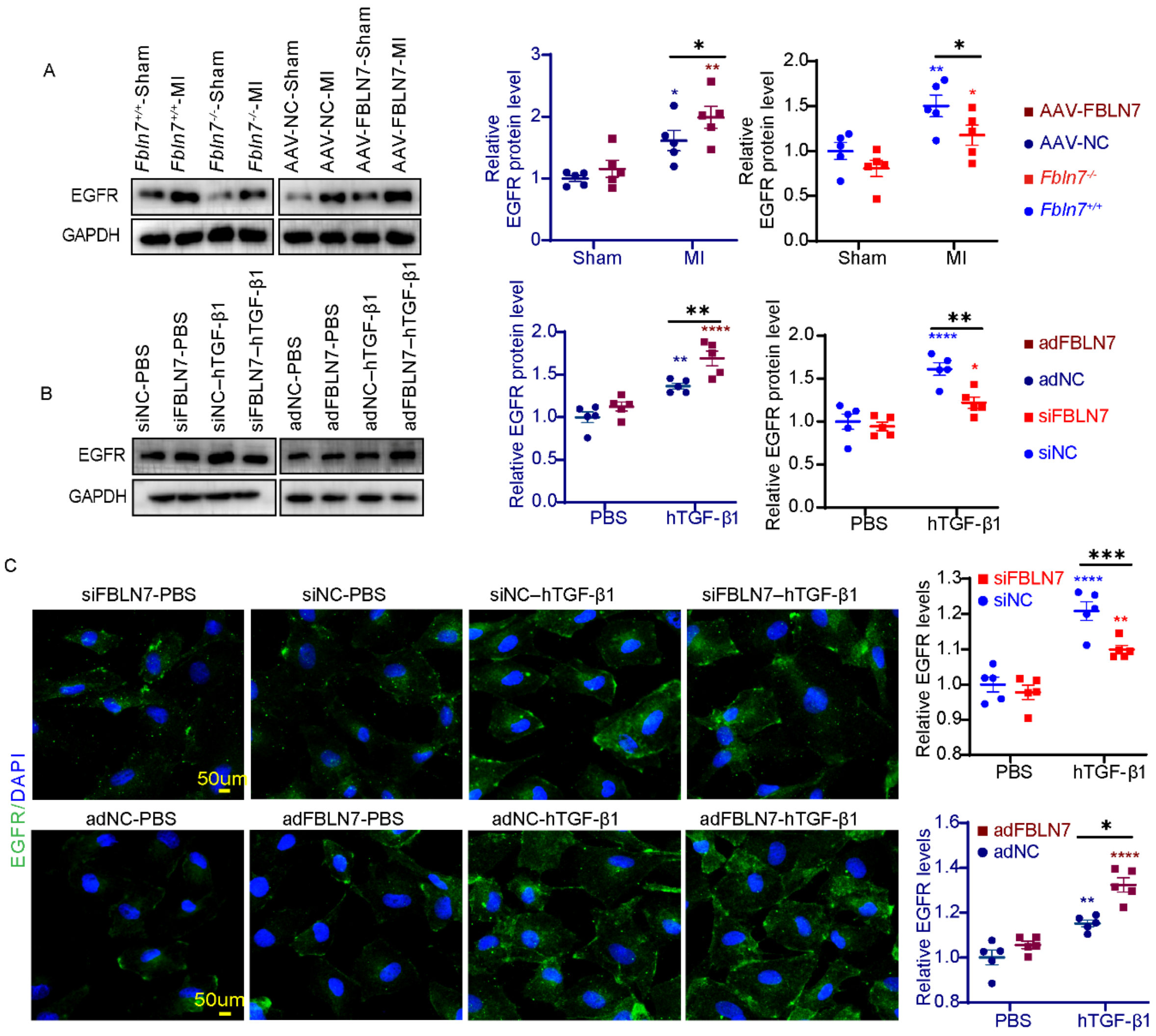

Figure S6. FBLN7 regulates EGFR protein level in heart tissues from MI mice and hTGF-β1–stimulated cardiac fibroblasts (CFs). A) Representative western blot images of EGFR protein level in heart tissues from FBLN7 KO (*Fbln7*$^{-/-}$) and overexpression (AAV-FBLN7) mice following MI or sham operation, and (B) in FBLN7 silencing (siFBLN7) and overexpression (adFBLN7) CFs after treatment with hTGF-β1 or PBS. GAPDH served as loading control. Quantifications of the band intensity were shown on the right. C) Representative immunofluorescence staining of EGFR protein in siFBLN7 and adFBLN7 cardiac fibroblasts (CFs) stimulated with hTGF-β1 or PBS. Quantifications were shown on the right. Quantifications are shown on the right. Error bars are SEM. *P<0.05, **P<0.01, ***P<0.001, ****P<0.0001 by two-way ANOVA followed by Sidak post hoc test. Red and blue asterisks, versus corresponding sham group.

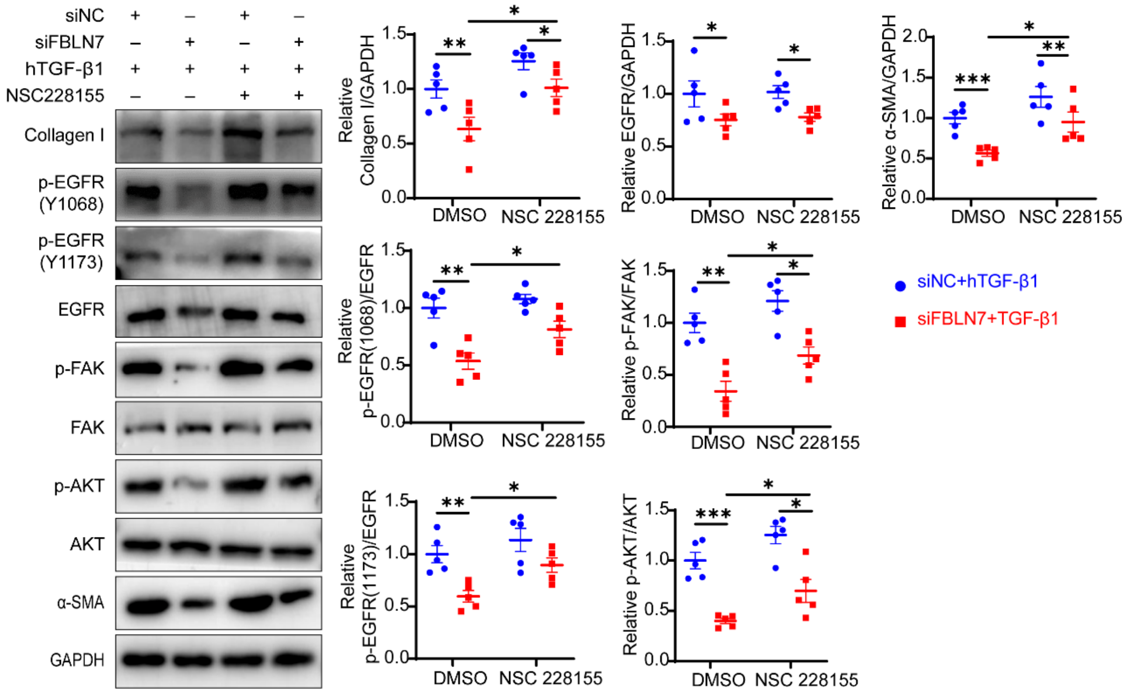

Figure S7. Effects of NSC 228155 on hTGF-β1–induced fibrotic responses in FBLN7-knockdown cardiac fibroblasts. Representative western blot images showing protein levels of p-EGFR(Y1068), p-EGFR(Y1173), EGFR, p-FAK, FAK, p-AKT, AKT, Collagen I, α-SMA and GAPDH in cardiac fibroblasts (CFs) transfected with siFBLN7 or siNC before treatment with TGF-β1 plus 0.8 uM NSC228155 (EGFR agonist) or TGF-β1 plus DMSO. Quantifications are shown on the right. Error bars are SEM. *P<0.05, **P<0.01, ***P<0.001, ****P<0.0001 by two-way ANOVA followed by Sidak post hoc test.

Table S1. Baseline characters of patients.

	Control (n=37)	Post-MI remodeling (n=39)	p value
Female (%)	27%	33%	0.55
Age (year)	62.27±1.70	64.62±1.99	0.10
SBP (mmhg)	124.1±3.40	110.3±4.48	0.04*
DBP (mmhg)	81.59±3.95	79.18±3.41	0.10
Hypertension (%)	65%	56%	0.45
Diabetes (%)	32%	36%	0.75
LVEF	0.65±0.01	0.34±0.02	P<0.001*
LVIDD (mm)	46.38±0.59	57.72±1.58	P<0.001*
FBLN7(ug/L)	0.99±0.07	1.61±0.11	P<0.001*

Data shown as Mean ± SEM, * P value < 0.05 was considered as significant.
SBP, systolic blood pressure. DBP, diastolic blood pressure. LVIDD, left ventricular internal diameter at diastole. LVEF, left ventricular ejection fraction.

Table S2. Polar interactions between FBLN7 polypeptide and EGFR.

Hydrogen bonds			
	Residues in FBLN7 polypeptide	Distance (Å)	Residues in EGFR
1	GLY 136[N]	2.88	ASP 347[OD1]
2	ILE 137[N]	2.86	ASP 347[OD1]
3	ASN 156[N]	3.02	ASP 347[OD2]
4	ARG 170[HH11]	2.16	HIS 383[O]
5	ARG 238[N]	3.30	ASN 444[OD1]
6	MET 241[N]	3.32	ARG 414[O]
7	CYS 244[N]	2.98	GLU 412[O]
8	THR 254[N]	3.48	ASN 413[OD1]
9	TYR 259[HH]	1.91	ASP 393[OD2]
10	CYS 268[SG]	3.35	ASN 413[OD1]
11	GLY 292[N]	3.23	GLN 390[O]
12	GLY 304[N]	3.41	ASN 36[OD1]
13	SER 305[N]	3.05	GLN 32[OE1]
14	CYS 145[SG]	3.36	HIS 383[NE2]
15	TYR 158[O]	3.04	THR 382[N]
16	CYS 160[SG]	3.02	HIS 383[NE2]
17	ASN 169[O]	3.47	SER 380[OG]
18	GLU 234[OE2]	2.28	LYS 467[HZ1]
19	HIS 242[O]	2.20	ASN 413[HD21]
20	TYR 259[OH]	1.87	ARG 414[HH21]
21	LYS 266[O]	3.20	ARG 414[N]
22	CYS 268[SG]	2.46	ASN 413[HD22]
23	GLU 269[O]	2.03	ARG 414[HH11]
24	ASP 270[OD2]	1.96	LYS 396[HZ1]
25	SER 309[OG]	2.36	ARG 309[HH11]
26	GLU 319[OE2]	3.49	ARG 309[N]
27	GLU 319[OE2]	3.31	ALA 310[N]
28	ARG 320[O]	3.20	ALA 310[N]

Salt bridges			
	Residues in FBLN7 polypeptide	Distance (Å)	Residues in EGFR
1	GLY 136[N]	2.88	ASP 347[OD1]
2	ARG 238[NE]	3.68	GLU 412[OE1]
3	GLU 234[OE1]	3.22	LYS 467[NZ]
4	GLU 234[OE2]	3.06	LYS 467[NZ]
5	ASP 270[OD2]	2.90	LYS 396[NZ]
6	GLU 300[OE2]	3.18	LYS 37[NZ]

Table S3. Antibodies and manufacturers.

Antibody	Company	Catalog No.	Concentrations
Fibulin-7	Bioss	bs-13161R	WB 1:1000; IHC 1:300; IF 1:100
Fibulin-7	Abcam	Ab151428	WB 1:1000
Collagen Type I	Proteintech	14695-1-AP	WB 1:2000; IHC 1:800

Collagen Type III	Abcam	ab7778	IHC 1:500
Collagen Type III	Abcam	ab184993	WB 1:1000
Anti-α SMA	Abcam	ab124964	WB 1:10000
Anti-α SMA	Abcam	ab7817	IF/ICC 1:500
Vimentin	Proteintech	60330-1-Ig	WB 1:10000
Vimentin	Abcam	ab8069	IF/ICC 1:200
p-Akt (Ser473)	CST	#4060	WB 1:1000
AKT	CST	#4691	WB 1:1000
p-FAK (Tyr397)	CST	#3283	WB 1:1000
FAK	CST	#3285	WB 1:1000
FAK	Proteintech	66258-1-Ig	IP 1:100
p-EGFR(Y1068)	Abcam	Ab40815	WB 1:2000
p-EGFR(Y1173)	Santa Cruz	Sc-57545	WB 1:200
EGFR	Abcam	Ab52894	WB 1:5000; IP 1:20
EGFR	Santa Cruz	sc-373746	ICC 1:50
GFP tag	Proteintech	66002-1-Ig	WB 1:50000; IP 1:200
MYC tag	Proteintech	16286-1-AP	WB 1:1000
His-Tag	Proteintech	66005-1-Ig	WB 1:20000; IP 1:200
Cardiac Troponin I	Proteintech	21652-1-AP	IF 1:200
Alpha Actinin	Proteintech	66895-1-Ig	IF 1:400
Anti-DDDDK tag	Abcam	Ab205606	IF 1:100
